# Proteins with RNA Chaperone Activity: A World of Diverse Proteins with a Common Task—Impediment of RNA Misfolding

**DOI:** 10.1155/2011/532908

**Published:** 2010-12-26

**Authors:** Katharina Semrad

**Affiliations:** Department of Biochemistry and Cell Biology, Max F. Perutz Laboratories, University of Vienna, Dr. Bohrgasse 9/5, 1030 Vienna, Austria

## Abstract

Proteins with RNA chaperone activity are ubiquitous proteins that play important roles in cellular mechanisms. They prevent RNA from misfolding by loosening misfolded structures without ATP consumption. RNA chaperone activity is studied *in vitro* and *in vivo* using oligonucleotide- or ribozyme-based assays. Due to their functional as well as structural diversity, a common chaperoning mechanism or universal motif has not yet been identified. A growing database of proteins with RNA chaperone activity has been established based on evaluation of chaperone activity via the described assays. Although the exact mechanism is not yet understood, it is more and more believed that disordered regions within proteins play an important role. This possible mechanism and which proteins were found to possess RNA chaperone activity are discussed here.

## 1. Introduction

Among all biological macromolecules, RNAs represent one of the most functionally versatile players in the cell. RNA molecules fulfill many different tasks such as coding and transfer of genetic information; they play regulatory functions in various cellular processes and catalyze chemical reactions (like cleavage and ligations). In addition to its functional versatility, RNAs are also able to fold into countless different structures, many of which have similar stabilities as the native structure and therefore compete with the native fold. Furthermore, RNA molecules often undergo transition states during their folding pathways before they reach the native and active structure. These transient structures can represent traps along the folding pathway from which the molecules might have a hard time to escape and which then end up being long-lived intermediates. The reason for this structural versatility is the fact that RNA consists of only four different bases which are easily capable of forming stable helices, that are not necessarily the native structure. The threshold for RNA molecules to be able to perform their functions is usually the accomplishment of reaching its native and active structure. 

In the cellular environment, RNA molecules do not appear as “naked” nucleic acids but always are found in conjunction with proteins. In some cases, the RNA molecule helps the protein partner to fold correctly; in others, the protein stabilizes the RNA structure. And last but not least proteins with RNA chaperone activity aid during the folding process of RNAs. Proteins with RNA chaperone activity open up misfolded RNA structures and do not require ATP [1]. Furthermore, after the RNA has been folded into its native structure, the protein becomes dispensable. Although the term “RNA chaperone” has been used routinely to describe various proteins that are capable to assist RNA folding *in vitro*, the term RNA chaperone is reserved to describe proteins whose RNA folding activity has been verified on its natural target RNA* in vivo*. Therefore, most of the proteins in this paper will be referred to as proteins with RNA chaperone activity if their RNA folding activity was only determined *in vitro* and/ or on nonnatural RNA targets. 

This paper will focus on the diversity of proteins with RNA chaperone activity and which experimental assays are in use to determine whether a protein has RNA chaperone activity. I will present examples for proteins with RNA chaperone activity and discuss possible mechanisms of RNA chaperoning. 

## 2. The Definition of Proteins with RNA Chaperone Activity and RNA Misfolding

### 2.1. What Are RNA Chaperones and Proteins with RNA Chaperone Activity?

The list of proteins with possible RNA chaperone activity is growing constantly. Proteins with different activities that support RNA folding are classified in this group. The definition of a protein with RNA chaperone activity is that the protein prevents RNA from misfolding by opening up misfolded structures. Proteins with RNA chaperone activity do not require ATP, which distinguishes them from RNA helicases, another group of proteins that facilitate RNA folding (e.g., Cyt-19) [2]. 

Proteins with RNA chaperone activity interact only transiently with RNA molecules and are supposed to be dispensable once the RNA has been folded correctly. This was shown for *E. coli* proteins S12 and StpA [3, 4]. A transient interaction and weak binding to RNA might be difficult to define because many of the identified RNA chaperones interact strongly with their target RNAs and are found in RNP complexes like ribosomal proteins, hnRNPs, La protein, and others. However, it has been demonstrated that a mutant StpA that shows stronger binding towards RNA shows decreased RNA chaperone activity suggesting that strong binding could also be detrimental to RNA folding [5]. In that way, proteins with RNA chaperone activity are also distinguished from “stabilizers” that are proteins that bind and stabilize an RNA structure and are required to stay bound in order to keep the RNA's native structure. Cyt-18, the tRNA synthetase from *Neurospora crassa*, is a “stabilizer” for the mitochondrial self-splicing group I intron: its presence is required to keep the native structure of the intron which otherwise unfolds readily. 

In the growing database of “proteins with RNA chaperone activity”, there exists an increasing number of proteins that simply possess RNA annealing activity. A prominent and intensively studied member of this group is the bacterial host factor Hfq that showed annealing activity on random substrates. Hfq in addition is an RNA chaperone as it was further demonstrated that Hfq does possess unwinding activity upon its native substrates [6, 7].

In brief, the group of proteins with RNA chaperone activity includes proteins that, first, open up misfolded structures without requirement of ATP and that, second, are dispensable once the RNA has been folded. 

### 2.2. RNA Misfolding

RNA molecules are prone to misfold *in vitro* and are usually prevented from misfolding *in vivo*. RNA basically encounters two folding problems: a kinetic folding problem, where the RNA molecule has to surmount kinetic barriers during the search for its native structure. Secondly, RNA molecules meet a thermodynamic folding problem as the final native structure often has to compete with alternative folds that have similar energetic stabilities [1]. RNA folding is a hierarchical process, and first secondary structure elements have to form. Secondary structural elements form between regions within the RNA molecule that are in close proximity. They are A-form helices consisting of Watson-Crick base-pairs. Secondary structures are very stable. The stability of a base-pair depends on the stability of both of its neighbouring base-pairs. Already any RNA of a reasonable length is able to form alternative base-pairs leading to alternative helices that become folding traps. 

Tertiary structures are higher order structures that are built by assembling the secondary structure elements into a more complex collapsed fold. They can also involve formation of helices. This is the case in pseudoknots where either a loop region interacts with a distant single stranded region or with another distant loop. Pseudoknots possess similar stability as secondary structures. But tertiary structural elements involve also other non-Watson-Crick interactions where for example, not only the Watson-Crick site of the nucleotide interacts with another nucleotide but also the Hoogsteen edge or the sugar edge of the nucleotide is involved in hydrogen bonding [8]. An often reoccurring tertiary structure motif is the A-minor interaction where an adenine interacts with the minor groove of the A-form helix [9]. Tertiary structures are often less stable and depend on the formation of secondary structures. Finally, monovalent or divalent metal ions play an important role in tertiary structure formation. 

The first studies on RNA structure and folding were done in the 1960s with yeast tRNA molecules. Already then it was demonstrated that tRNAs are able to adopt two distinct conformations of which only one is the native structure which can be aminoacylated [10, 11]. 

The RNA folding problem becomes even more prominent in the case of large RNAs such as group I introns or in the context of large protein-ribonucleic acid complexes such as RNase P and the ribosome. 

It was demonstrated that the self-splicing group I intron of the thymidylate synthase gene of phage T4 misfolds in the absence of translation: when the ribosome does not prevent base-pairing between exon and intron sequences, the intron is not able to fold correctly and cannot perform the splicing reaction [12]. A similar observation was made with the group I intron of *Tetrahymena thermophila* ribosomal RNA: a subset of molecules misfolds and accumulates into an inactive population [13]. Misfolding depends on exon sequences that form stable hairpins and intervene with 5′-splice-site formation. 

*In vivo*, however, some group I introns require the assistance of proteins to splice efficiently and prevent misfolding. For an example, the Cyt-18 protein in *Neurospora crassa* mitochondira is a tRNA synthetase which stabilizes the P4-P6 domain of group I introns and recruits Cyt-19, an RNA helicase, which then unwinds folding traps and promotes splicing [2, 14].

For large RNP complexes such as the ribosome, a growing body of evidence suggests that several additional factors such as helicases exist that assist during the folding process* in vivo*. 

## 3. Assays to Measure RNA Chaperone Activity

As proteins with RNA chaperone activity are very heterogeneous concerning their structure and their way to resolve the folding of RNA molecules, there are various RNA chaperone assays available to measure different activities. In principle, the assays can be divided into* in vitro* and* in vivo* assays that use either simple oligonucleotide annealing or displacement reactions or that measure catalytic activities of correctly folded ribozymes. 

Measuring an activity that might be targeted more specifically to a certain subset of substrates in the natural environment of the putative RNA chaperone makes it difficult to evaluate RNA chaperone activity using a single assay. The substrates in the* in vitro* assay (e.g., oligonucleotides) might differ in sequence requirements or structure requirements from possible native substrates and might lead to negative results. Strand unwinding assays might give positive results in the case of single-strand binding proteins. Furthermore, *in vivo* assays to measure RNA chaperone activity can be negatively influenced by possible toxicity of the putative RNA chaperone when overexpressed or can lead to secondary effects in the cell that give false positive results. Therefore, it is recommended to measure the RNA chaperone activity at least in more than one chaperone assay to be certain that no non-specific activity is measured. 

### 3.1. In Vitro RNA Chaperone Activity Assays (see Figure 1)

#### 3.1.1. Oligonucleotide Annealing

In this assay, two complementary oligonucleotides (oligos) present in concentrations above their dissociation constant are incubated together in the absence and in the presence of the protein to be evaluated for RNA chaperone activity. An increase in the rate of duplex formation is observed when the tested protein has RNA annealing activity. In principle two complementary short RNA oligos are used in this assay, although it has been a habit to choose DNA oligos instead. In order to measure RNA chaperone activity, however, I think that RNA oligonucleotides should be the preferred choice. Detection methods of duplex formation include native gel electrophoresis where the two complementary RNA strands can either be visualized by radioactive or fluorophor labelling and has been used in (besides many many other publications) [15–17]. RNA annealing can alternatively be measured by observing the fluorescence resonance energy transfer (FRET) upon the closing up of the two fluorescently labelled oligonucleotides (e.g., see [18, 19]).

#### 3.1.2. Oligonucleotide Melting and Strand-Displacement Activities

The ability of a protein to open up and unwind an already formed RNA duplex is measured. To measure RNA chaperone activity in contrast to helicase activity, this assay is performed in the absence of ATP or an alternative energy source. Analogous to the annealing assays, melting activity can be detected by using native gel electrophoresis or by measuring loss of fluorescence energy resonance transfer (FRET) that occurs upon dissociation of the complementary fluorophor labelled RNAs. In addition to the above-described detection methods, new approaches to detect folding or unfolding of single molecules emerge and include time-resolved NMR, which becomes a powerful tool to study folding of small RNAs. 

#### 3.1.3. Hammerhead Ribozyme Cleavage

The hammerhead ribozyme cleavage reaction and folding of the ribozyme-substrate 3-way helical junction have been studied in a great detail. Therefore, this assay represents a suitable tool to study RNA chaperone activity upon folding of the hammerhead ribozyme-substrate construct. Using the hammerhead cleavage assay, both annealing and strand displacement can be studied independently of each other [3, 20]. The advantage on the well-studied assay is that depending whether single-turnover conditions or multiple turnover conditions are employed, it is possible to distinguish between annealing and strand dissociation activities. Using single-turnover conditions, where an excess of ribozyme and low concentrations of substrate are applied, substrate annealing is determined as substrate annealing becomes the rate limiting step. On the other hand, using multiple turnover conditions with an excess of substrate over ribozyme, the whole cleavage reaction is monitored consisting of annealing and product release. Since product release represents the rate limiting step, product dissociation is measured. 

#### 3.1.4. Group I Intron Splicing

Self-splicing of the thymidylate synthase group I intron (td intron) of bacteriophage T4 has been characterized, and the td intron has been used lengthily to monitor RNA chaperone activity of various proteins. Splicing of different group I intron constructs that do not fold readily into the splicing competent structure *in vitro* is tested with and without chaperones.

**Cis-Splicing Assay**

In this *td* intron construct both, 5′ and 3′ exons are shortened for the upstream exon down to 27 nucleotides and the downstream exon shortened to only 2 nucleotides. This short exon construct (td shosho) splices at 37°C but RNA chaperones increase folding and as a consequence the splicing rate of the short-exon construct is increased as well [5].

**Trans-Splicing Assay**

Here, the td intron is split into two halves in the center of loop L6 in the P4-P6 domain, where in the wild type group I intron an open reading frame for an endonuclease is present. The upstream *in vitro* transcribed construct contains 549 nucleotides of exon1 and 131 nucleotides of the 5′-part of the intron. The downstream construct consists of the remaining 147 nucleotides of the intron and 23 nucleotides of exon2. Correct and efficient folding of the trans-intron-constructs is significantly impaired at 37°C but works fine at elevated temperatures (55°C), which is monitored through splicing [21]. Chaperones with strong annealing and unwinding activities such as ribosomal protein L1 or L19 from E.coli are capable to catalyze trans-splicing at 37°C or even at lower temperatures, for example, hnRNPI increases splicing at 25°C [22].

### 3.2. In Vivo RNA Chaperone Activity Assays (see Figure 2)

#### 3.2.1. In Vivo Folding Trap Assay in *E. Coli*

Splicing of the group I intron within the thymidylate synthase gene of phage T4 occurs efficiently* in vivo*. Though, when splicing and translation are uncoupled by introducing stop codons in the upstream exon, splicing is significantly reduced. This is due to alternative base-pairing of exonic and intronic sequences which prevent the formation of the intron's native fold [12]. The mutant td precursor construct tdSH1 consists of an exonic stop codon and has an additional intronic point mutation (C865U) which further destabilizes the native intron structure. The tdSH1 construct is significantly impaired in splicing *in vivo*. Overexpression of RNA chaperones in the presence of the tdSH1 mutant is used to evaluate if the RNA chaperone is able to rescue the misfolded intron and restore splicing [23]. 

#### 3.2.2. Transcription Antitermination Assay in *E. Coli*

Transcription read-through of the chloramphenicol acetyl transferase gene (cat) is inhibited due to the preceding transcription terminator stem. The stable hairpin secondary structure of the terminator inhibits the polymerase to transcribe the cat gene and as a consequence no chaloramphenicol resistance is achieved. The cells are chaloramphenicol (Cm) sensitive. Proteins with RNA chaperone activity are able to melt the terminator stem and as a consequence read-through occurs and the cells become chloramphenicol resistant. The transcription antitermination assay was used for assaying cold shock proteins or IF1 from *E. coli * [24–26]. 

## 4. Proteins with RNA Chaperone Activity

Proteins that possess RNA chaperone activity are very divers and span from viral to bacterial and human proteins that are involved in many different cellular processes. The list of RNA chaperones is permanently growing. Recently, a database for proteins with RNA chaperone activity was established where every lab is able to contribute their data for a newly identified chaperone http://www.projects.mfpl.ac.at/rnachaperones/index.html [27]. The following selection of proteins with RNA chaperone activity is not complete but points out the most important groups or single proteins.

### 4.1. Virus-Encoded RNA Chaperones

The first viral RNA chaperone activities were reported in the early 1990s and showed that nucleocapsid protein 7 (Ncp7) of HIV increases hammerhead ribozyme cleavage significantly [20, 28]. Only a few years later, another virus-encoded protein with RNA chaperone activity has been identified, the HDV delta antigen, which was also monitored in the hammerhead ribozyme assay and furthermore shown to be dispensable after folding has occurred [29]. 

Flaviviridae core proteins were also monitored and shown to possess RNA chaperone activity in a hammerhead cleavage assay and/or RNA strand annealing activities [30, 31]. Interestingly, many of the viral proteins show exceptional high degree of disorder. In the case of the Flaviviridae core proteins, it was also reported that heat denaturation still retained strand annealing activity suggesting that the disordered domains of the proteins are involved in chaperoning. 

Nucleocapsid proteins from two members of the Coronaviridae family have been investigated and hammerhead ribozyme cleavage was shown to be enhanced in their presence [32, 33]. And again both nucleocapsid proteins show a high degree of disorder in in silico predictions. 

A growing body of evidence suggests that there exist many more viral proteins that possess RNA chaperone activity. The list here is not complete but proteins that were shown to have only DNA annealing activities were left out in this list. The majority of these small viral proteins show strong propensity for disorder which suggests that disorder might be a mechanistic requirement for chaperoning.

Interestingly, studies on Nc proteins demonstrated that these proteins not only possess RNA chaperone activity *in vitro* but also are required for strand annealing and strand displacement activities on their target RNAs *in vivo *[33]. Recently, a specific template switching assay designed to study strand displacement in a retroviral-derived system demonstrated that nucleocapsid protein from Coronavirus shows RNA chaperone activity and most likely is an RNA chaperone *in vivo * [34].

### 4.2. StpA

The *E. coli* transcriptional regulator StpA, a 15 kD basic protein, was isolated as a repressor of a splicing-deficient group I intron in thymidylate synthase of phage T4 [4]. StpA was furthermore shown to possess strong RNA chaperone activity *in vivo* in the folding trap assay [35]. The protein was tested *in vitro* in a strand-annealing and strand-displacement assay and exhibited strong activities in both tests [36]. More detailed studies on StpA revealed that the protein binds transiently to RNA with a preference for unstructured regions and that binding to RNA is diminished in the presence of high ionic strength [5]. In an elaborate study applying *in vivo* DMS modifications to the RNA,* in vivo* folding of the group I intron was evaluated in the absence and presence of StpA [37]: Schroeder and coworkers demonstrated that StpA opens up tertiary interactions of the td group I intron. While the loosening effect is advantageous in wild type or misfolded introns, overexpression of StpA in the presence of introns that were already destabilized in their 3D structure was detrimental. Structure prediction of StpA suggested that this protein exhibits more than 70% disorder and it was suggested that this unfolded regions of StpA might play a role in chaperoning [38]. 

### 4.3. Ribosomal Proteins

Ribosomal proteins are required within every cellular organism to build up the bacterial 70S or the eukaryal 80S ribosome. Many ribosomal proteins further regulate transcription or translation of their own operons. In addition, ribosomal proteins are also involved in various very different cellular processes and fulfill extraribosomal functions [39, 40]. Ribosomal proteins are highly conserved among various species and many ribosomal proteins have unusual long unstructured extensions that wind their way through the ribosome [41]. 

The first observation that ribosomal proteins are capable of chaperoning RNA folding came from the Belfort lab: screening for cellular factors that increase trans-splicing of the thymidylate synthase group I intron revealed that many ribosomal proteins possess chaperoning activity, with ribosomal protein S12 from the small ribosomal subunit having the strongest activity [3]. Furthermore, S12 significantly increased hammerhead ribozyme cleavage [3]. A systematic study on large ribosomal subunit proteins from *E. coli* showed that 1/3 of the tested proteins possesses strong RNA chaperone activity *in vitro* in the trans-splicing assay [21]. In addition, ribosomal protein L1 orthologs from eukarya, bacteria, and mesophilic archaea also exhibited strong trans-splicing and cis-splicing activities* in vitro* [42]. Although it makes sense that the RNA chaperone activity of ribosomal proteins could play a role during ribosome assembly, a definite proof for the requirement of this activity *in vivo* has not yet been provided. Recently, it was demonstrated that *E.coli *ribosomal proteins L15, L16, L18, and L19, that showed RNA chaperone activity* in vitro*, further possess protein chaperone activity comparable to other protein chaperones such as Hsp90 [43]. It was suggested that intrinsically unstructured domains of ribosomal proteins could play a role in chaperoning. The exact mechanism, however, still remains elusive (see Section 5). 

### 4.4. Cold Shock Proteins and IF1

Cold shock proteins (csps) are conserved throughout bacteria and plants. They are expressed during cold-shock, when misfolding of RNAs becomes a major problem for the organism and function as transcriptional antiterminator at low temperature. Many experiments that have been performed to describe chaperone activity of cold-shock proteins utilize DNA helices (and only sometimes in addition RNA duplexes) and refer to the activity as nucleic acid melting activity. However, it has to be mentioned that there are no elaborate studies on whether there is a difference between RNA duplex and DNA duplex melting and whether DNA melting activity automatically is the same as RNA melting. 

*E. coli* contains nine members of the csp family and CspA, the major cold-shock protein and CspE were identified to interact non-specifically with RNA molecules and to possess nucleic acid melting activities [44–46]. 

Cold-shock proteins in higher plants are highly conserved. Glycine-rich and Zn-finger containing proteins from *Arabidopsis thaliana* have been monitored for their nucleic acid melting activity, and it was shown that GRP7 (glycine-rich protein) and CSDP1 (cold shock domain protein) possess RNA chaperone activity [47]. A recent study also demonstrated that out of six glycine-rich proteins in rice (*Oryza sativa*), which are likely to be involved in adaptation to cold-shock, three of them exhibit RNA- (and DNA-) melting activities suggesting that GRPs in plants fulfill a chaperoning role during low temperatures [48]. 

In *E. coli* translation, initiation factor 1 (IF1) is a small 71 amino-acid long peptide, which contains 5 rigid *β*-barrels and belongs to the OB (oligomer-binding)-fold proteins such as the cold shock proteins. N- and C-termini of IF1 are highly flexible. It was demonstrated that *E. coli* IF1 is capable of complementing for a cspB and cspC double knock-out in *Bacillus subtilis* suggesting that IF1 and csps have at least partially overlapping activities [49]. *E. coli* IF1 exhibits RNA chaperone activity in various assays including RNA annealing of complementary oligonucleotides, trans-splicing, *in vivo* folding trap assay, and transcription anti-termination *in vivo* and *in vitro* [25, 50]. 

### 4.5. hnRNPs and Human La Protein

Heteronuclear Ribonucleoproteins encompass a group of about 20 polypeptides that are predominantly nuclear in localization and are involved in RNA processing. 

The first observation that hnRNPs possess RNA chaperone activity came in the early 1990s when fractionated HeLa nuclear extract was tested for annealing activity of an mRNA and its antisense partner. Three proteins, hnRNP A1, C1 and U, were identified and hnRNP A1 was further shown to enhance hammerhead ribozyme cleavage *in vitro* [16, 20]. Later, a detailed study on possible functions of Ro RNPs, which are Ro ribonucleoprotein complexes, composed of a small noncoding cytoplasmic RNA, termed Y RNA and its protein partners was conducted: besides the permanently associated proteins Ro60 and La, subpopulations of Ro-RNPs also contain hnRNP I and hnRNP K, both of which exhibited strong RNA chaperone activity *in vitro *in the trans-splicing and the cis-splicing assay [22]. hnRNP I is identical to poly-pyrimidine binding protein (PTB) isoform 4 and was identified as a splicing suppressor in mammalian cells [51]. It regulates cap-independent translation, localization of cytoplasmic RNAs, and poly-A-site cleavage [52]. PTB belongs to the IRES transacting factors (ITAFs), which are host factors (like La, hnRNP K, nucleolin, unr and many others) that interact with viral RNAs and induce conformational changes that then lead to translation initiation [53]. It was further reported that calcivirus replication requires PTB but only at lower or at higher temperatures than the permissive 37°C, suggesting a chaperoning role of PTB [54]. Members of the group of ITAFs have been implicated in RNA chaperoning like unr, a cold-shock domain containing protein [55], human La protein, and hnRNP K [22].

HnRNP K is also a multifunctional protein that is a transcriptional factor for c-myc and c-src [56–58]; it enhances splicing [59] and is a translational regulator [60]. 

La proteins primarily bind RNA polymerase III transcripts and protect them from nuclease attack [61]. They also interact with pre-tRNAs at their UUU-3′OH ends and facilitate their maturation. La contributes to assembly of RNP complexes by retaining RNAs in the nucleus. La is also involved in translation regulation. And human La was demonstrated to possess RNA chaperone activity *in vitro* in the cis-splicing assay and *in vivo* in the folding trap assay [22]. 

### 4.6. Hfq

The bacterial protein Hfq was first discovered in the end 1960s as a host factor for bacteriophage Q*β* replication [62]. The bacterial protein is a pleiotropic regulator for gene expression in bacteria. It interacts with many small RNAs and their mRNA targets and regulates posttranscriptional regulation of small noncoding RNAs such as DsrA, sodB, oxyS, rprA, and spot42 [63–67]. Hfq preferentially binds to A/U rich, unstructured regions. 

Hfq encompasses an Sm-domain, which is highly conserved among various species and usually is found in eukaryotic spliceosomal RNPs. Crystallographic studies of *Escherichia coli*, *Pseudomonas aeruginosa,* and *Staphylococcus aureus* Hfq proteins showed that Hfq forms homohexameric ring structures with a central cationic pore that forms the RNA binding site [68–70]. 

Using strand annealing and strand melting assays to measure RNA chaperone activity of Hfq, only strand annealing activity was observed [27]. However, a detailed study using RNase footprinting on Hfq's interaction with its target RNAs sodB mRNA and the small noncoding regulator ryhB RNA demonstrated that Hfq indeed did loosen secondary structures within sodB mRNA that lead to binding of its regulatory RNA rhyB [7]. A similar observation was made using fluorescence labelled rpoS mRNA and dsrA small noncoding RNA [6]. By means of FRET, it was shown that Hfq induces annealing of dsrA to rpoS mRNA and prior to the annealing event Hfq disrupts rpoS secondary structure elements. Consequently, Hfq is entitled to be called RNA chaperone.

### 4.7. Human Chaperones in Disease

#### 4.7.1. Prion Protein

The prion protein is a misfolded isoform of the essential component of prion diseases such as Creutzfeldt-Jakob disease in humans—one of several neurodegenerative diseases. The function of the human prion protein is not clearly understood. It was demonstrated that the prion protein has RNA (and DNA) annealing activity [71]; however, it was not yet shown if it possesses also RNA unwinding activity and may therefore be classified as an “annealer”. Interestingly, the prion protein contains an intrinsically unstructured N-terminal domain [72]. 

#### 4.7.2. Fragile X Mental Retardation Protein (FMRP)

The fragile X mental retardation protein (FMRP) is linked to the fragile X syndrome as the disease is due to transcriptional silencing of the gene. FMRP possesses RNA binding activity and its interaction partners include a large number of mRNAs, microRNAs, siRNAs, and small noncoding RNAs as well as a multitude of different proteins [73–76]. It was demonstrated using hammerhead ribozyme cleavage that FMRP possesses RNA chaperone activity [77]. And finally in line with many other proteins with RNA chaperone activity, it is interesting to mention that FMRP consists of a highly disordered C-terminus suggesting that the substrate versatility of FMRP might be accomplished through its structural disorder [78].

## 5. Mechanisms

Chaperones provide a critical cellular activity. Proteins with RNA chaperone activity are very divers in structure as well as in function: StpA, a transcriptional activator and repressor of a multitude of bacterial genes, is a small (15 kD) bacterial protein with intrinsically unstructured regions. StpA has strong RNA chaperone activity. On the other hand the bacterial protein Hfq is a large multidomain protein complex (60 kD) and folds into a compact ring-like structure. Among ribosomal proteins, many were shown to possess RNA chaperone activity (e.g., one third of large ribosomal subunit proteins from *Escherichia coli* show RNA chaperone activity *in vitro*). Ribosomal proteins are usually small proteins many of which have long unstructured domains and are highly basic proteins.

**Figure 3:**
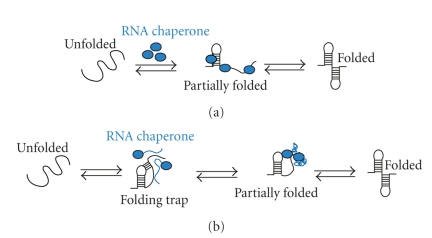
*Hypothetical mechanisms of RNA chaperoning.* (a) shows folding of an RNA molecule in the presence of RNA chaperones (blue). RNA chaperones and proteins with RNA chaperone activity prevent the RNA from misfolding and increase annealing of the correct structure by crowding. (b) Proteins with RNA chaperone activity possessing disordered regions (blue) interact with misfolded RNA. Upon energy transfer, the RNA structure loosens and the disordered protein domain becomes more ordered. Proteins with RNA chaperone activity are dispensable in both cases after the RNA has folded into its native form.

Proteins with RNA chaperone activity do not require an external energy source as RNA helicases do. This raises the question of how RNA chaperones accomplish the RNA folding task and where the energy for this process comes from. Proteins with RNA chaperone activity in most cases encompass two major activities: the annealing activity and the unwinding activity (see also Figure 3). Many proteins with RNA chaperone activity are highly basic proteins and therefore interact readily with negatively charged RNA molecules. In that way, they might stabilize folded states by bringing together distant regions of the RNA molecule and as a consequence increase RNA double-strand formation. This mechanism could be comparable to the action of chemical chaperones such as osmolytes which are small organic compounds, that do not interfere with the cellular metabolism but speed up folding processes enabled through a crowding effect [79]. 

Another indication that a crowding effect might play a role at least to some extent during RNA annealing is the following: when RNA chaperone activity is measured *in vitro*, there is always an excess of protein over RNA present in the assay. For example, in the trans-splicing assay, 200 nMols of RNAs (leading to a 20 nM end-concentration) are tested for folding in the presence of 1-2 *μ*M protein. It was shown that *E. coli* ribosomal protein L1 displays maximal RNA chaperone activity starting from 400 nM up to 2 *μ*M protein concentration [42]. This means that at least a 20-fold excess of protein to RNA has to be present to achieve maximal chaperoning activity of ribosomal protein L1 from *E. coli*. 

In this line, it also has to be mentioned that in the* in vivo* chaperone assay, which uses the folding trap of a misfolded group I intron in the thymidylate synthase gene of phage T4, it is always necessary that the measured protein is overexpressed and available in higher concentrations [23]. For example, the *E. coli* protein StpA, which is found constitutively expressed in the bacterial cell, only shows its RNA chaperone activity *in vivo* when StpA is additionally over-expressed from an expression vector, thus showing that the cellular concentration of StpA is not sufficient to increase folding of the misfolded group I intron. Certainly, this observation might be due to the engagement of StpA in other regulatory functions in the bacterial cell; however, it also points to the direction that more than one molecule of StpA is required to assist folding of the td group I intron. As a consequence the question rises if and how it is possible to distinguish between RNA chaperone activity and a nonspecific single-strand RNA binding activity of the protein that might both prevent misfolding. Using the *in vivo* folding trap assay, however, not only proteins with possible RNA chaperone activity like StpA had been tested but also a viral single strand binding protein from Influenza virus (NP) was tested and did not show any increase in splicing suggesting that single strand RNA binding might not be sufficient for chaperoning. Furthermore, a detailed study on StpA wild type and mutants demonstrated that only the full-length StpA was able to show RNA chaperone activity by simultaneously interacting with two RNA molecules [5]. 

RNA chaperone activity of StpA has been studied for more than a decade. It was shown that StpA has strong *in vivo* and *in vitro *RNA chaperone activities. In a mechanistical *in vivo* study of StpA, Schroeder and coworkers demonstrated that StpA loosens tertiary contacts within the thymidylate synthase group I intron [37]. In contrast, the *Neurospora crassa* tRNA synthetase Cyt-18 that also increases group I intron splicing of td stabilizes tertiary interactions. But how is the opening of tertiary structure elements accomplished without the hydrolysis of ATP? This strand unwinding activity is more difficult to explain as the question remains of how a protein can actively open up hydrogen bonds when no apparent source of energy is required. 

In the protein world, it became more and more visible that the classical structure-function paradigm does not necessarily hold for many proteins and their activities. A growing body of evidence suggests that a multitude of proteins do not fold into compact domains but are fully or at least partially unstructured [80]. In eukaryotes, for example, conservative estimations point out that 5%–15% of all proteins are completely disordered and 50% of the cellular proteins have at least long unstructured domains. An interesting study by Tompa and Csermely demonstrated that among chaperones a significantly high percentage of proteins show long unstructured regions [38]. Among RNA chaperones, the percentage of at least partially disordered proteins is even higher (54%) than in the group of protein chaperones (36.7%). Disordered proteins and protein segments allow a broad versatility for interaction partners and in this case for interaction with different RNA molecules. But it can also explain the ability of proteins with RNA chaperone activity to multitask as so far no RNA chaperone has been identified whose only task is to aid in RNA folding. Interestingly, it was recently demonstrated that some ribosomal proteins that possess RNA chaperone activity and contain disordered regions are also capable to chaperone protein folding suggesting once again that disordered regions provide high versatility for substrate interactions [43]. 

The idea of disordered RNA chaperones is especially attractive because there are many advantages of proteins with disordered regions over compact proteins: (1) the main advantage of a disordered region is that it can easily interact with a range of many different partners and is not limited to a single binding pocket or recognition element on a partner molecule. (2) The bigger surface of the unstructured protein might provide a “loosening effect” for the incorrectly folded RNA molecule. (3) The troublesome question of where the energy for the RNA unwinding might come from could be explained by the gain of compactness upon interaction with the RNA and a simultaneous loosening of the RNA structure (see Figure 3(b)). As a consequence, the RNA gains another chance to fold correctly. (4) The intrinsically unstructured protein might provide a folding platform for the RNA as the chaperone holds the RNA molecule in close proximity.

## 6. Outlook

In future the research focus on RNA chaperones will lie on the understanding of the molecular mechanism and how intrinsically unstructured regions in proteins might play a role in function. Interestingly, two very closely related ribosomal proteins L1 from Archaea, that encompass 70% aminoacid identity, possess opposite activities: the mesophilic L1 protein displays strong RNA chaperone activity whereas the thermophilic one inhibits ribozyme assays [42]. A detailed mutation study will likely shed light on the different activities and explain the RNA chaperoning mechanism at least for a subgroup of RNA chaperones. 

The big challenge, however, will be to identify *in vivo* targets of RNA chaperones. RNA chaperones are evaluated in assays for their broad specificity but *in vivo* they might be specialized to supervise folding of only a subset of RNA molecules. The specificity might possibly be conferred by a different domain than the chaperoning activity. 

## Figures and Tables

**Figure 1:**
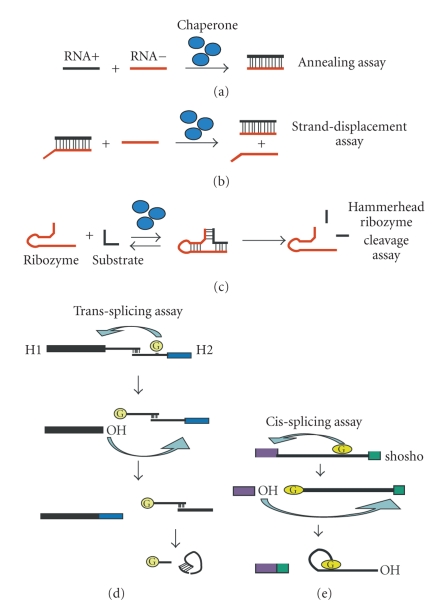
*In vitro* chaperone assays. (a) shows a simple annealing assay where two complementary RNA strands are annealed in the presence of RNA chaperones. (b) In the strand displacement assay, the RNA duplex is loosened and an alternative RNA helix is formed. (c) Hammerhead ribozyme cleavage is enhanced in the presence of chaperones. Under single turnover conditions, substrate to ribozyme annealing is measured. Under multiple turnover conditions substrate dissociation is measured. (d) In the trans-splicing assay, the group I intron is split in two halves H1 (upstream exon, 5′-part of the intron) and H2 (3′-part of the intron and exon2), and splicing at low temperatures in the presence of chaperones is measured. (e) shows the cis-splicing assay where an enhancement of splicing at 37°C is measured in the presence of chaperones. The construct (shosho) contains short exon 1 (27 nucleotides) and short exon 2 (2 nucleotides) sequences.

**Figure 2:**
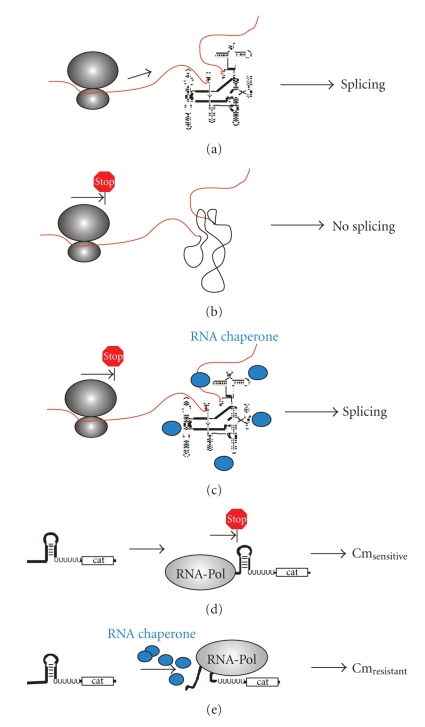
*In vivo* chaperone assays. (a–c) show the *in vivo* folding trap assay: (a) In the presence of translation, the group I intron folds correctly. (b) In the absence of translation, misfolding of the group I intron occurs. (c) Proteins with RNA chaperone activity loosen misfolded structures and splicing can proceed. (d–e) show the *in vivo* antitranscription termination assay. (d) The transcription terminator stem folds and transcription of the chloramphenicol acetyl transferase cannot proceed. Thus, cells are chloramphenicol sensitive. (e) Proteins with RNA chaperone activity loosen the terminator stem, transcription can occur, and the cells become chloramphenicol resistant.

## References

[1] Herschlag D (1995). RNA chaperones and the RNA folding problem. *The Journal of Biological Chemistry*.

[2] Mohr S, Stryker JM, Lambowitz AM (2002). A DEAD-Box protein functions as an ATP-dependent RNA chaperone in group I intron splicing. *Cell*.

[3] Coetzee T, Herschlag D, Belfort M (1994). *Escherichia coli* proteins, including ribosomal protein S12, facilitate in vitro splicing of phage T4 introns by acting as RNA chaperones. *Genes and Development*.

[4] Zhang A, Derbyshire V, Galloway Salvo JL, Belfort M (1995). *Escherichia coli* protein StpA stimulates self-splicing by promoting RNA assembly in vitro. *RNA*.

[5] Mayer O, Rajkowitsch L, Lorenz C, Konrat R, Schroeder R (2007). RNA chaperone activity and RNA-binding properties of the *E. coli* protein StpA. *Nucleic Acids Research*.

[6] Arluison V, Hohng S, Roy R, Pellegrini O, Régnier P, Ha T (2007). Spectroscopic observation of RNA chaperone activities of Hfq in post-transcriptional regulation by a small non-coding RNA. *Nucleic Acids Research*.

[7] Geissmann TA, Touati D (2004). Hfq, a new chaperoning role: binding to messenger RNA determines access for small RNA regulator. *EMBO Journal*.

[8] Leontis NB, Westhof E (2001). Geometric nomenclature and classification of RNA base pairs. *RNA*.

[9] Nissen P, Ippolito JA, Ban N, Moore PB, Steitz TA (2001). RNA tertiary interactions in the large ribosomal subunit: the A-minor motif. *Proceedings of the National Academy of Sciences of the United States of America*.

[10] Gartland WJ, Sueoka N (1966). Two interconvertible forms of tryptophanyl sRNA in E. coli. *Proceedings of the National Academy of Sciences of the United States of America*.

[11] Lindahl T, Adams A (1966). Native and renatured transfer ribonucleic acid. *Science*.

[12] Semrad K, Schroeder R (1998). A ribosomal function is necessary for efficient splicing of the T4 phage thymidylate synthase intron in vivo. *Genes and Development*.

[13] Woodson SA, Cech TR (1991). Alternative secondary structures in the 5′ exon affect both forward and reverse self-splicing of the Tetrahymena intervening sequence RNA. *Biochemistry*.

[14] Caprara MG, Lehnert V, Lambowitz AM, Westhof E (1996). A tyrosyl-tRNA synthetase recognizes a conserved tRNA-like structural motif in the group I intron catalytic core. *Cell*.

[15] Cusick ME, Belfort M (1998). Domain structure and RNA annealing activity of the *Escherichia coli* regulatory protein StpA. *Molecular Microbiology*.

[16] Portman DS, Dreyfuss G (1994). RNA annealing activities in HeLa nuclei. *EMBO Journal*.

[17] Urbaneja MA, Wu M, Casas-Finet JR, Karpel RL (2002). HIV-1 nucleocapsid protein as a nucleic acid chaperone: spectroscopic study of its helix-destabilizing properties, structural binding specificity, and annealing activity. *Journal of Molecular Biology*.

[18] Rajkowitsch L, Schroeder R (2007). Coupling RNA annealing and strand displacement: a FRET-based microplate reader assay for RNA chaperone activity. *BioTechniques*.

[19] Rajkowitsch L, Schroeder R (2007). Dissecting RNA chaperone activity. *RNA*.

[20] Herschlag D, Khosla M, Tsuchihashi Z, Karpel RL (1994). An RNA chaperone activity of non-specific RNA binding proteins in hammerhead ribozyme catalysis. *EMBO Journal*.

[21] Semrad K, Green R, Schroeder R (2004). RNA chaperone activity of large ribosomal subunit proteins from *Escherichia coli*. *RNA*.

[22] Belisova A, Semrad K, Mayer O (2005). RNA chaperone activity of protein components of human Ro RNPs. *RNA*.

[23] Prenninger S, Schroeder R, Semrad K (2006). Assaying RNA chaperone activity in vivo in bacteria using a ribozyme folding trap. *Nature Protocols*.

[24] Bae W, Xia B, Inouye M, Severinov K (2000). *Escherichia coli* CspA-family RNA chaperones are transcription antiterminators. *Proceedings of the National Academy of Sciences of the United States of America*.

[25] Phadtare S, Kazakov T, Bubunenko M, Court DL, Pestova T, Severinov K (2007). Transcription antitermination by translation initiation factor IF1. *Journal of Bacteriology*.

[26] Phadtare S, Severinov K, Inouye M (2003). Assay of transcription antitermination by proteins of the CspA family. *Methods in Enzymology*.

[27] Rajkowitsch L, Chen D, Stampfl S (2007). RNA chaperones, RNA annealers and RNA helicases. *RNA Biology*.

[28] Tsuchihashi Z, Khosla M, Herschlag D (1993). Protein enhancement of hammerhead ribozyme catalysis. *Science*.

[29] Huang ZS, Wu HN (1998). Identification and characterization of the RNA chaperone activity of hepatitis delta antigen peptides. *The Journal of Biological Chemistry*.

[30] Cristofari G, Ivanyi-Nagy R, Gabus C (2004). The hepatitis C virus Core protein is a potent nucleic acid chaperone that directs dimerization of the viral (+) strand RNA in vitro. *Nucleic Acids Research*.

[31] Ivanyi-Nagy R, Lavergne JP, Gabus C, Ficheux D, Darlix JL (2008). RNA chaperoning and intrinsic disorder in the core proteins of Flaviviridae. *Nucleic Acids Research*.

[32] Zúñiga S, Sola I, Cruz JLG, Enjuanes L (2009). Role of RNA chaperones in virus replication. *Virus Research*.

[33] Zúñiga S, Sola I, Moreno JL, Sabella P, Plana-Durán J, Enjuanes L (2007). Coronavirus nucleocapsid protein is an RNA chaperone. *Virology*.

[34] Zúñiga S, Cruz JLG, Sola I, Mateos-Gómez PA, Palacio L, Enjuanes L (2010). Coronavirus nucleocapsid protein facilitates template switching and is required for efficient transcription. *Journal of Virology*.

[35] Clodi E, Semrad K, Schroeder R (1999). Assaying RNA chaperone activity in vivo using a novel RNA folding trap. *EMBO Journal*.

[36] Rajkowitsch L, Semrad K, Mayer O, Schroeder R (2005). Assays for the RNA chaperone activity of proteins. *Biochemical Society Transactions*.

[37] Waldsich C, Grossberger R, Schroeder R (2002). RNA chaperone StpA loosens interactions of the tertiary structure in the td group I intron in vivo. *Genes and Development*.

[38] Tompa P, Csermely P (2004). The role of structural disorder in the function of RNA and protein chaperones. *FASEB Journal*.

[39] Warner JR, McIntosh KB (2009). How common are extraribosomal functions of ribosomal proteins?. *Molecular Cell*.

[40] Wool IG (1996). Extraribosomal functions of ribosomal proteins. *Trends in Biochemical Sciences*.

[41] Ramakrishnan V, Moore PB (2001). Atomic structures at last: the ribosome in 2000. *Current Opinion in Structural Biology*.

[42] Ameres SL, Shcherbakov D, Nikonova E, Piendl W, Schroeder R, Semrad K (2007). RNA chaperone activity of L1 ribosomal proteins: phylogenetic conservation and splicing inhibition. *Nucleic Acids Research*.

[43] Kovacs D, Rakacs M, Agoston B (2009). Janus chaperones: assistance of both RNA- and protein-folding by ribosomal proteins. *FEBS Letters*.

[44] Jiang W, Hou Y, Inouye M (1997). CspA, the major cold-shock protein of *Escherichia coli*, is an RNA chaperone. *The Journal of Biological Chemistry*.

[45] Jones PG, Mitta M, Kim Y, Jiang W, Inouye M (1996). Cold shock induces a major ribosomal-associated protein that unwinds double-stranded RNA in *Escherichia coli*. *Proceedings of the National Academy of Sciences of the United States of America*.

[46] Phadtare S, Severinov K (2005). Nucleic acid melting by *Escherichia coli* CspE. *Nucleic Acids Research*.

[47] Kim JS, Park SJ, Kwak KJ (2007). Cold shock domain proteins and glycine-rich RNA-binding proteins from Arabidopsis thaliana can promote the cold adaptation process in *Escherichia coli*. *Nucleic Acids Research*.

[48] Kim JY, Kim WY, Kwak KJ, Oh SH, Han YS, Kang H (2010). Glycine-rich RNA-binding proteins are functionally conserved in arabidopsis thaliana and oryza sativa during cold adaptation process. *Journal of Experimental Botany*.

[49] Weber MHW, Beckering CL, Marahiel MA (2001). Complementation of cold shock proteins by translation initiation factor IF1 in vivo. *Journal of Bacteriology*.

[50] Croitoru V, Semrad K, Prenninger S (2006). RNA chaperone activity of translation initiation factor IF1. *Biochimie*.

[51] Lin CH, Patton JG (1995). Regulation of alternative 3′ splice site selection by constitutive splicing factors. *RNA*.

[52] Wagner EJ, Garcia-Blanco MA (2001). Minireview: polypyrimidine tract binding protein antagonizes exon definition. *Molecular and Cellular Biology*.

[53] Stoneley M, Willis AE (2004). Cellular internal ribosome entry segments: structures, trans-acting factors and regulation of gene expression. *Oncogene*.

[54] Karakasiliotis I, Chaudhry Y, Roberts LO, Goodfellow IG (2006). Feline calicivirus replication: requirement for polypyrimidine tract-binding protien is temperature-dependent. *Journal of General Virology*.

[55] Mitchell SA, Spriggs KA, Coldwell MJ, Jackson RJ, Willis AE (2003). The Apaf-1 internal ribosome entry segment attains the correct structural conformation for function via interactions with PTB and unr. *Molecular Cell*.

[56] Michelotti EF, Michelotti GA, Aronsohn AI, Levens D (1996). Heterogeneous nuclear ribonucleoprotein K is a transcription factor. *Molecular and Cellular Biology*.

[57] Ritchie SA, Pasha MK, Batten DJP (2003). Identification of the SRC pyrimidine-binding protein (SPy) as hnRNP K: implications in the regulation of SRC1A transcription. *Nucleic Acids Research*.

[58] Takimoto M, Tomonaga T, Matunis M (1993). Specific binding of heterogeneous ribonucleoprotein particle protein K to the human c-myc promoter, in vitro. *The Journal of Biological Chemistry*.

[59] Expert-Bezancon A, Caer JPL, Marie J (2002). Heterogeneous nuclear ribonucleoprotein (hnRNP) K is a component of an intronic splicing enhancer complex that activates the splicing of the alternative exon 6A from chicken *β*-tropomyosin pre-mRNA. *The Journal of Biological Chemistry*.

[60] Ostareck DH, Ostareck-Lederer A, Wilm M, Thiele BJ, Mann M, Hentze MW (1997). mRNA silencing in erythroid differentiation: hnRNP K and hnRNP E1 regulate 15-lipoxygenase translation from the 3′ end. *Cell*.

[61] Wolin SL, Cedervall T (2002). The La protein. *Annual Review of Biochemistry*.

[62] Franze De Fernandez MT, Eoyang L, August JT (1968). Factor fraction required for the synthesis of bacteriophage Q*β*-RNA. *Nature*.

[63] Massé E, Gottesman S (2002). A small RNA regulates the expression of genes involved in iron metabolism in *Escherichia coli*. *Proceedings of the National Academy of Sciences of the United States of America*.

[64] Massé E, Majdalani N, Gottesman S (2003). Regulatory roles for small RNAs in bacteria. *Current Opinion in Microbiology*.

[65] Moller T, Franch T, Hojrup P (2002). Hfq: a bacterial Sm-like protein that mediates RNA-RNA interaction. *Molecular Cell*.

[66] Sledjeski DD, Whitman C, Zhang A (2001). Hfq is necessary for regulation by the untranslated RNA DsrA. *Journal of Bacteriology*.

[67] Zhang A, Wassarman KM, Ortega J, Steven AC, Storz G (2002). The Sm-like Hfq protein increases OxyS RNA interaction with target mRNAs. *Molecular Cell*.

[68] Nikulin A, Stolboushkina E, Perederina A (2005). Structure of Pseudomonas aeruginosa Hfq protein. *Acta Crystallographica Section D*.

[69] Sauter C, Basquin J, Suck D (2003). Sm-like proteins in Eubacteria: the crystal structure of the Hfq protein from *Escherichia coli*. *Nucleic Acids Research*.

[70] Schumacher MA, Pearson RF, Møller T, Valentin-Hansen P, Brennan RG (2002). Structures of the pleiotropic translational regulator Hfq and an Hfq-RNA complex: a bacterial Sm-like protein. *EMBO Journal*.

[71] Gabus C, Derrington E, Leblanc P (2001). The prion protein has RNA binding and chaperoning properties characteristic of nucleocapsid protein NCP7 of HIV-1. *The Journal of Biological Chemistry*.

[72] Marc D, Mercey R, Lantier F (2007). Scavenger, transducer, RNA chaperone? What ligands of the prion protein teach us about its function. *Cellular and Molecular Life Sciences*.

[73] Brown V, Jin P, Ceman S (2001). Microarray identification of FMRP-associated brain mRNAs and altered mRNA translational profiles in fragile X syndrome. *Cell*.

[74] Ishizuka A, Siomi MC, Siomi H (2002). A Drosophila fragile X protein interacts with components of RNAi and ribosomal proteins. *Genes and Development*.

[75] Jin P, Alisch RS, Warren ST (2004). RNA and microRNAs in fragile X mental retardation. *Nature Cell Biology*.

[76] Miyashiro KY, Beckel-Mitchener A, Purk TP (2003). RNA cargoes associating with FMRP reveal deficits in cellular functioning in Fmr1 null mice. *Neuron*.

[77] Gabus C, Mazroui R, Tremblay S, Khandjian EW, Darlix JL (2004). The fragile X mental retardation protein has nucleic acid chaperone properties. *Nucleic Acids Research*.

[78] Ivanyi-Nagy R, Davidovic L, Khandjian EW, Darlix JL (2005). Disordered RNA chaperone proteins: from functions to disease. *Cellular and Molecular Life Sciences*.

[79] Somero GN (1986). Protons, osmolytes, and fitness of internal milieu for protein function. *The American Journal of Physiology*.

[80] Wright PE, Dyson HJ (1999). Intrinsically unstructured proteins: re-assessing the protein structure-function paradigm. *Journal of Molecular Biology*.

